# “Dead” Exciton Layer and Exciton Anisotropy of Bulk MoS_2_ Extracted from Optical Measurements

**DOI:** 10.1021/acsnano.2c07169

**Published:** 2022-11-09

**Authors:** Vasyl G. Kravets, Alexander A. Zhukov, Matthew Holwill, Kostya S. Novoselov, Alexander N. Grigorenko

**Affiliations:** † Department of Physics and Astronomy, Manchester University, Manchester M13 9PL, United Kingdom; ‡ National Graphene Institute, Manchester University, Manchester M13 9PL, United Kingdom; § Institute for Functional Intelligent Materials, National University of Singapore, 117544, Singapore

**Keywords:** excitons, optics, anisotropy, dead layer, layered materials

## Abstract

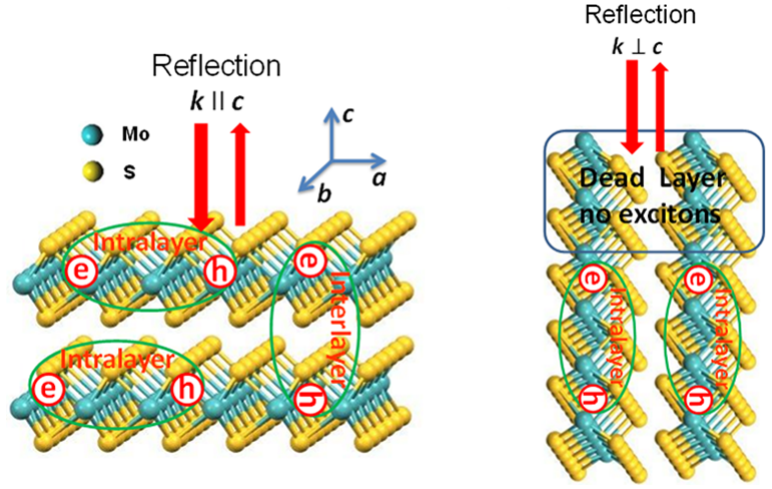

Excitons (electron–hole pairs bound by the Coulomb potential) play an important role in optical and electronic properties of layered materials. They can be used to modulate light with high frequencies due to the optical Pauli blocking. The properties of excitons in 2D materials are extremely anisotropic. However, due to nanometre sizes of excitons and their short life times, reliable tools to study this anisotropy are lacking. Here, we show how direct optical reflection measurements can be used to evaluate anisotropy of excitons in transition metal dichalcogenides MoS_2_. Using focused beam spectroscopic ellipsometry, we have measured the polarized optical reflection of bulk MoS_2_ for two crystal orientations: *c*-axis being perpendicular to the surface from which reflection is measured and *c*-axis being parallel to the surface from which reflection is measured. We found that for the parallel configuration the optical reflection near excitonic transitions is strongly affected by the presence of the exciton “dead” layer such that the excitonic reflection peaks become the excitonic dips due to light interference. At the same time, the optical reflection for the perpendicular orientation is not significantly altered by the exciton “dead” layer due to large anisotropy of exciton properties. Performing simultaneous Fresnel fitting for both geometries, we were able to evaluate exciton anisotropy in layered materials from simple optical measurements.

Recently discovered family of layered materials (LMs) could establish a foundation for a next generation of nanophotonics. LMs possess extremely large refractive indices and high anisotropy useful for nanophotonics applications.^1−3^ Some of their optical properties can be modulated by external electric fields,^4^ providing a path to compact light modulators.^5^ Furthermore, optical nonlinearity could be very pronounced in LMs.^6^ Hence, study of optoelectronic properties of LMs presents an important and useful task. One of the most interesting features of LMs is the presence of excitons produced by electron–hole pairs bound by the Coulomb potential. It is the excitons that allow light modulation due to the Pauli blocking effect as well as various electronic applications. Owing to the layered nature of LM, the properties of excitons in anisotropic LM materials are extremely anisotropic. However, due to the nanometer sizes of excitons and their short life times, it is difficult to study the exciton anisotropy in LMs.

It is worth noting that optical anisotropy of LMs reveals a fundamental relationship between structural and optoelectronic properties. The optical properties of LMs originate from light emission by in-plane and out-of-plane dipoles.^7,8^ A particular class of LMs—transition metal dichalcogenides (TMDCs)—recently attracted a lot of attention due to their optical and electronics properties, which provide an exciting possibility for the creation of various components of nano-optics.^9^ In TMDCs, excitons become dipole emitters and contribute to optical features in the visible light.^3,10^ Optical excitations in the TMDC family of materials are normally understood in terms of Wannier excitons and trions.^11^ Interband and excitonic transitions in TMDCs have a large optical strength for the in-plane orientation of the dipole, but transitions associated with out-of-plane polarization are strongly affected by the quantum confinement of electronic wave functions in a small region around the plane. Excitons in TMDCs (and LMs in general) can be divided into two types according to whether the electron and hole always reside on the same layer (intralayer) or can be found on different layers (interlayer).^7,8^ Hence, the exciton dipole moment is highly anisotropic, with the dipole’s strength and orientation dictated by the particular electronic properties of the material, its dipole selection rules, and local-field effects.^12,13^

The unusual optical properties of excitons in TMDCs come about from the electronic structure, which assumes different behavior of electrons moving along the layers and in the perpendicular direction. These properties are normally probed by measuring polarized optical reflection spectra. However, such spectra could be strongly affected by some excitonic transitional layer (which could increase its width near excitonic transitions as compared to that observed outside the excitonic range). Historically, Hopfield and Thomas^14^ suggested the theory of spatial dispersion in LMs, which described the shape and amplitude of polarized reflectance in the spectral range of exciton excitations and the disappearance of reflectance at exciton energies for special orientation of the sample and selected polarization of light. They showed that the exciton dipole moment tends to zero near the sample surface for the case of large-sized Wannier excitons, implying the presence of a surface transitional layer where no exciton can be located. This region was later termed as the exciton-free “dead layer”.^15^ Being a transitional surface layer, it has optical properties different from the bulk, and its thickness is defined by the spatial dimensions of excitons (which could be much larger than the atomic spacing). Hopfield’s theory^14^ was later applied to understand the behavior of excitons in GaAs^16^ and CdS.^15^ It was found that the presence of the dead layer could change reflection drops to reflection peaks due to light interference at the excitonic transitions.^15^ Spectral optical measurements were employed to investigate the properties of these “vanishing” excitons through observation of excitons near surface edges^17,18^ and flat nanosheets,^19^ while extremely high optical anisotropy in MoS_2_ (which could lead to high exciton anisotropy) was observed by the means of spectral ellipsometry.^3,20,21^ The presence of the exciton-free dead layer (the transitional layer near the surface with optical properties different to the bulk) made the extraction of anisotropic optical constants of TMDCs a very tricky task, despite the great importance and interest in excitons in TMDC materials.

This task is inherently connected to another important problem. Indeed, the same way as 2D atomic materials are often different from their respective bulk counterparts (*e*.*g*., 2D graphene possesses massless Dirac electrons in contrast to 3D graphite,^22^ 2D MoS_2_ is a direct semiconductor while 3D MoS_2_ is indirect one,^23^*etc*.), the optical properties of the surface layers of 3D materials are often completely different from the optical properties of the corresponding bulk materials. This implies that there exists a transitional layer near the surface (of any optical material!) with optical properties different to those of the bulk. This transitional layer is conditioned by the fact that the atomic layers end at the surface, where the crystal lacks translational and inversion symmetries (hence, the crystal arrangement near the surface is different from that of the bulk) and is normally several atomic layers thick. Generally, the transitional surface layer does not affect the optical response of 3D optical materials very much unless for some singular cases, such as the Brewster phenomenon.^24^ However, the high refractive index and high anisotropy of LMs could make the contribution of the transitional surface layer significant such that it could affect the optical response of the samples, leading to ambiguity in measuring optical constants of LMs using standard Fresnel theory.^25^ In addition, the presence of optically active excitons in LMs could significantly increase the size of this transitional layer and strongly affect optical properties of the samples.

To address all these difficulties, here, we study the optical reflection from bulk MoS_2_ crystals for two crystals orientations: *c*-axis being perpendicular to the crystal surface from which reflection was measured and *c*-axis being parallel to the surface from which reflection was measured. We use spectroscopic ellipsometry to measure angle-resolved polarized reflection. We show that the spectral optical reflection from the parallel crystal orientation cannot be described by simple Fresnel theory due to the presence of the exciton-free dead transitional surface layer. This implies that care is needed when extracting optical constants of TMDCs (and LMs in general) from spectral optical measurements. Moreover, this also implies that exciton anisotropy of LMs could be evaluated from simple optical reflection measurements. We provide an additional confirmation of anisotropic characteristics of excitons in MoS_2_ based on the measured Jones–Mueller matrix components. We also provide direct observation of excitation of in-plane and out-of-plane excitons by light of different polarizations (which was previously observed only for photoluminescence). Our observation of the surface excitons in the layered MoS_2_ for in-plane or out-of-plane geometries has direct analogy with the Frenkel excitons in organic semiconductors.^26,27^ Finally, the anisotropic refractive index of MoS_2_ extracted from our measurements can be useful for engineering various photonic devices, such as waveguides, light absorbers, and light emitters based on TMDs.

## Results and Discussion

### Optical Properties of the Samples

**Figure 1:**
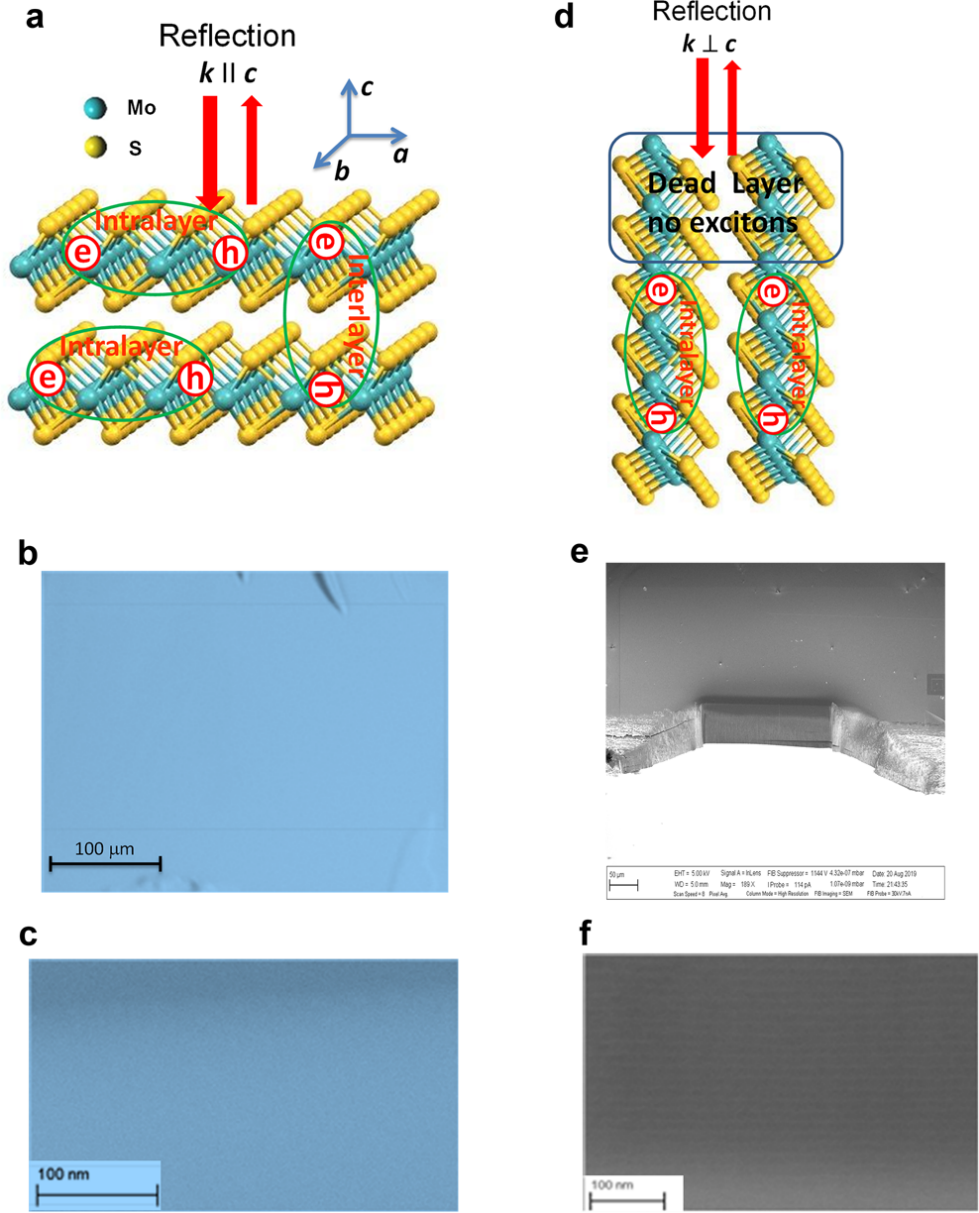
Schematic design of experiment for the two configurations of direction of incident light respect to *c*-axis of stacked MoS_2_ sheets and optical and SEM images. Left panel for horizontally stacked MoS_2_ sheets: (a) Schematics of the reflectance measurements and existence of intralayer and interlayer excitons. (b and c) Optical and SEM images of the surface of layered MoS_2_ sheets. Right panel for vertically stacked MoS_2_ sheets: (d) schematic of the reflectance measurements and existence of intralayer excitons with additional dead layer on top of surface corresponding to Hopfield’s model. (e) *In situ* SEM image at θ = 54° tilt used for milling sample. (f) SEM microscope image of polished surface of sample after final FIB milling.

We measured anisotropic optical properties of MoS_2_ crystals in two different crystal orientations: Geometry 1 (G1), where *c*-axis was perpendicular to the surface from which light reflection was measured, and Geometry 2 (G2), where *c*-axis was parallel to the surface from which reflection was measured (see schematics in Figure 1, and details in the Methods section). Layered MoS_2_ crystals are built from S–Mo–S units bonded by van der Waals forces. Each of these stable units consists of two hexagonal planes of S atoms sandwiching a hexagonal plane of Mo coordinated through ionic–covalent interactions with each other in a trigonal prismatic arrangement, as shown in Figure 1a,d (see refs (9 and 28)). It is worth noting that G1 is the standard geometry normally produced by transfer of MoS_2_ flakes onto a substrate. It allows one to probe in-plane optical constants. G2 was achieved by cutting the edge of the MoS_2_ flake and using its edge for the measurements. This gives an access to the out-of-plane optical response of the crystal. A typical example of the sample used for G2 is presented in Figure 1e,f and Figure S1, Supporting Information (SI). The scanning electron microscopy (SEM) image of the *ac*-plane from the freshly cleaved crystal after ions milling and polishing reveals a local smooth surface (Figure 1e,f), which ensures that our measurements are reliable with the studied area of more than 100 × 200 μm^2^ large enough to get reliable light reflection coefficient in the focused spectroscopic measurements. Light interaction with MoS_2_ crystals is schematically shown in Figure 1a,d: for G1, the direction of incident light wavevector ***k*** was parallel to *c*-axis of the MoS_2_ hexagonal planes; for G2, the direction of incident wavevector ***k*** was perpendicular to *c*-axis. For each geometry, the polarized spectroscopic reflection was measured at the normal angle of incidence: electric field ***E*** was directed for G1 as ***E*** ⊥ ***c*** with two orientations 0° and 90° degrees, which lead to *p*- and *s*-polarizations under angle measurements, respectively; and for G2 as ***E*** ⊥ ***c*** and ***E*** || ***c*** (while ***k*** ⊥ ***c***).

**Figure 2:**
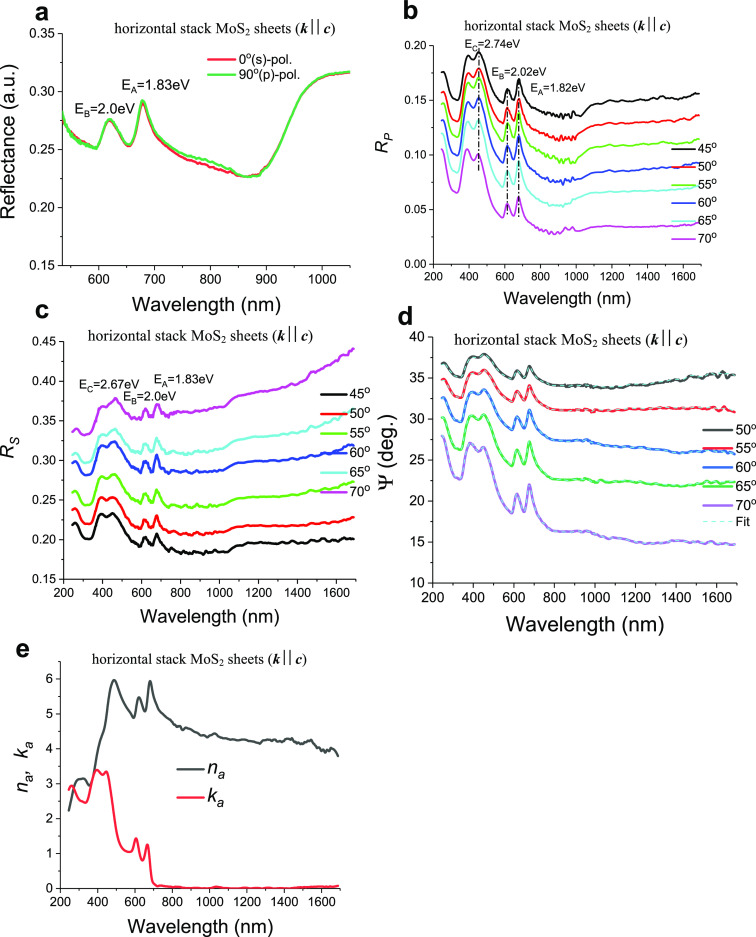
Reflectance and ellipsometric spectra of horizontally stacked MoS_2_ sheets. (a) Reflectance spectra at normal incidence for two polarizations of incident light. (b and c) Features of reflection for *p-* and *s-*polarized light as a function of incident angles contributed from the absorbance of the A and B excitonic bands and interband transition. (d) Experimental and modeled dependences of the ellipsometric parameter Ψ at various angles of incidence. (e) Complex refractive index extracted from spectroscopic ellipsometric data.

**Figure 3:**
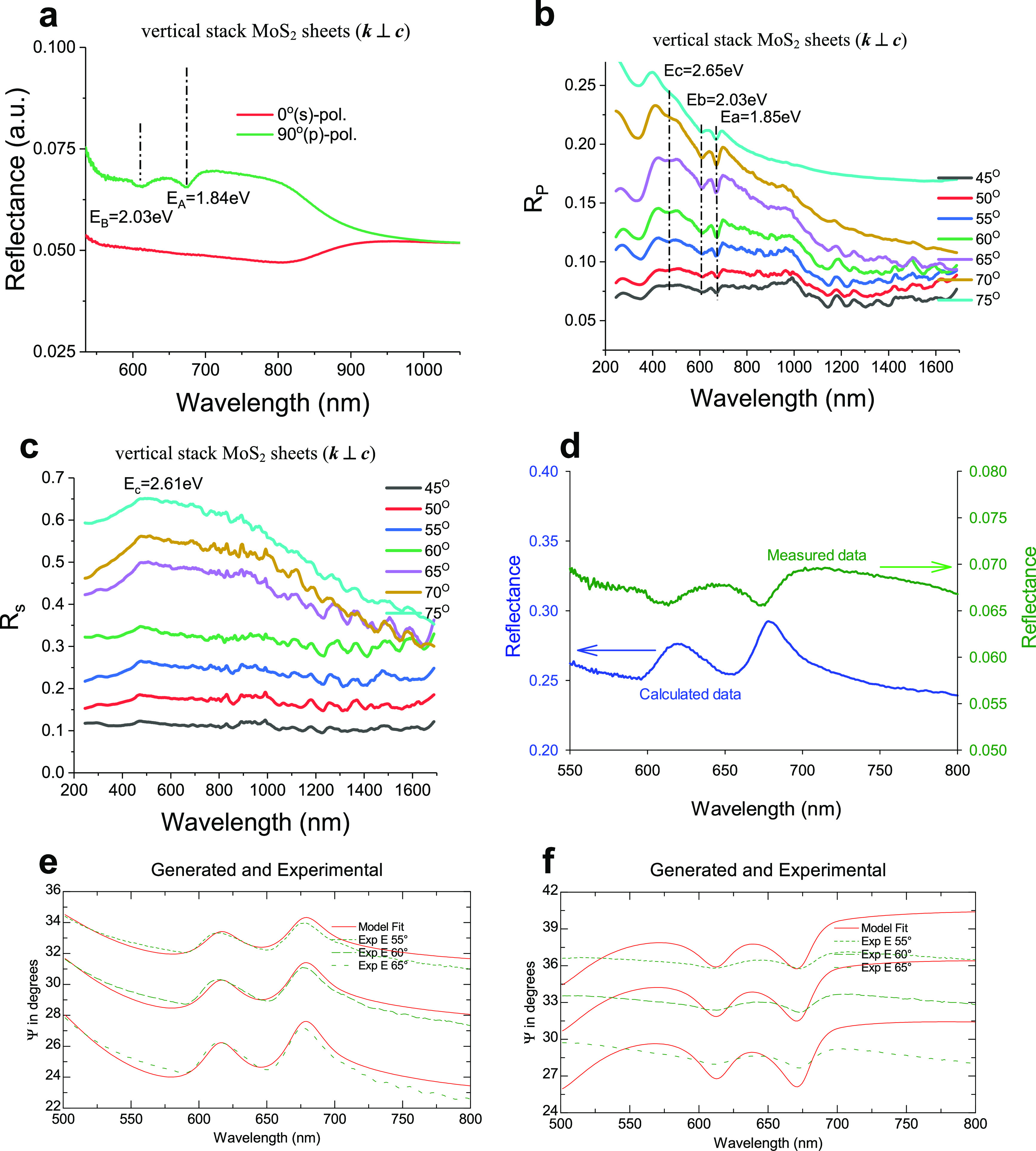
Reflectance and ellipsometric spectra of vertically stacked MoS_2_ sheets. (a) Reflectance spectra at normal incidence for two polarizations of incident light. (b and c) Features of reflection for *p-* and *s-*polarized light as a function of incident angles contributed from the absorbance of the A and B excitonic bands and interband transition. (d) Experimental (the green curve) and modeled (the blue curve) dependences of normal reflection for 90° polarization. (e) Fitting of the measured ellipsometric spectra in G1 geometry with a dead layer of 1 nm. (f) Joint fitting of the measured ellipsometric spectra in G2 geometry with a dead layer of 16 nm.

The reflectivity *R*(λ) was measured in the range 500–1100 nm with respect to the reference spectrum of a Ag thick mirror at normal angle of incidence using a Fourier transform spectrometer (Figures 2a and 3a and Figure S2, (SI)). For G1 geometry (Figure 2a), both 0°- and 90°-polarization spectra exhibit semiconductor behavior, characterized by the features in the region of excitonic absorption A (1.83 eV) and B (2.0 eV) (see Figure 2a). Since the reflection spectra for both polarizations mostly coincide, we conclude that the in-plane anisotropy (the difference between *a* and *b* axes) is not large.

The observed resonances A and B arise from transitions between the 2-fold split valence band and the conduction band around the K point of the Brillouin zone.^23^ The angle/wavelength dependences of polarized *R*_p,s_(λ) spectra measured with the help of focused beam spectroscopic ellipsometer (shown in Figure 2b,c) are characterized by four peaks around the so-called A (1.82 eV), B (2.02 eV), and C (2.74 eV) excitonic resonances and D resonance (3.1 eV), in agreement with previous studies based on thick, few-layer and monolayer MoS_2_.^29−32^ The ellipsometric function Ψ (which describes polarized reflection, see the Methods section) also shows prominent peaks near the exciton resonances that are almost independent of the angle of incidence (Figure 2d).

### Optical Modeling

To model the measured ellipsometric data, we initially applied an isotropic model for a thick MoS_2_ layer with the aim to extract approximate values of the optical constants n* = n + ik. The fitting of the ellipsometry data was performed using Woollam WVASE32 software in which the complex refractive index of a flat unknown layer can be extracted using a fitting procedure based on the Fresnel theory. After performing such a fitting procedure (which gave a reasonable fit, see Figure 2d), we then used the anisotropic biaxial model for the thick MoS_2_ layer. The same good fit between the measured and modeled ellipsometric spectra was observed for either isotropic or the anisotropic model (Figure 2d). Such a low sensitivity of ellipsometric spectra to the *c*-components of the complex refractive index of stacked 2D MoS_2_ multilayers, *n*_a_* = n_*a*_ + *ik*_*a*_ and *n*_c_* = n_*c*_ + *ik*_*c*_, is due to a large in-plane refractive index *n*_*a*_ ∼ 4 (Figure 2e). According to the Snell’s law for the incident light at 50°–75°, this gives the refraction angles of only ∼11°–14°, which implies that the probed electric field of the refracted light mainly concentrates along the *c*-axis and, thus, is almost insensitive to the out-of-plane dielectric component.^3^ Previous analyses of the spectra of optical constants *n*_a_* = n_*a*_*+ ik*_*a*_ show that main features are associated with A and B excitons due to the transition from the spin–orbit split valence bands to the lowest conduction band at the *K* and *K’* points in the low-energy region, while the C and D peaks result from the transition electrons between the valence and conduction bands at the Λ and Γ points^29,31,32^ part of the Brillouin zone.

In order to extract the complex refractive index for out-of-plane (*ac*) components from ellipsometric measurements (in addition to the in-plane (*ab*) constant extracted with Fresnel fitting of the reflection data for G1), we used G2 arrangement. Hence, we moved to the configuration where *c*-axis is parallel to the sample surface (Figure 1d). This geometry provided us with two important observations. First, the normal reflectance spectra show quenching of the contribution of the A and B excitons for 0°-polarized light (see Figure 3a) (0°-polarized light here corresponds to the case where electric field of the incident light is parallel to *c*-axis and ***k*** ⊥ ***c***; it would evolve to *s*-polarization at some small angle). Second, unexpectedly, we observed an inversion of the exciton A and B peaks observed for G1 (which are shown in Figure 2a) into dips for 90°-polarized light observed for G2 (shown in Figure 3a). The exciton energies of the dips for 90°-polarization are slightly shifted to higher values: A (1.84 eV), B (2.03 eV), and C (2.61 eV) (compare Figures 2a and 3a).

The comparison of reflection spectra, *R*_p_(λ), measured at different angles (Figures 2b and 3b) confirms the shift of exciton features from 1.82 to 1.85 eV (681 to 670 nm) and 2.02–2.03 eV (614–611 nm) for the two studied geometries G1 and G2, respectively. It is worth reminding that, for G1, the shape of *p*-polarized angled reflectivity spectra are similar to those obtained under *s-*polarization (Figure 2b,c). This is due to the fact that refractive index is large, and hence, both *p-* and *s-*polarization for G1 have electric fields mostly in the in-plane direction. For G2, the situation is different. For *p*-polarized light reflection measured at different angles, we observe spectral dips in the photon energy range 1.5–3.8 eV, which are obviously related to the A and B excitonic dips in the range 1.8–2.1 eV shown in Figure 3a. At the same time, only a broad absorption shoulder at ∼2.61 eV appears for the *s*-polarized reflection spectra (Figure 3c) with no contribution from A and B excitons.

Fresnel modeling of the measured reflections in G2 orientation provided important insights. Using the anisotropic optical constants extracted in G1 orientation (which were reported in ref (3)) and anisotropic biaxial model of the Wvase32 software, we have modeled the optical properties of MoS_2_ for the edge measurements (G2). The modeled spectra for 0° polarization were in agreement with the measurements showing the absence of excitonic peaks. However, all modeled spectra for 90° polarization showed the excitonic peaks instead of excitonic dips observed in experiments. As an example, Figure 3d shows the normal reflectance in 90° polarization measured (the green curve) and the reflectance calculated with anisotropic Fresnel theory (the blue curve). One can see that the calculated reflectance is much larger than that measured in our experiments and that it predicts excitonic peaks instead of excitonic dips observed in our measurements. This disagreement (dips vs peaks) was observed for all calculated and measured data for *p*-reflection *R*_p_ and spectroscopic reflection Ψ.

In accordance with the Hopfield model described above, we have added the dead exciton layer for the two geometries (G1 and G2) in Fresnel modeling. Fortunately, Wvase32 software allows for simultaneous fit of simulated response to measured data in both geometries. This fit is shown in Figure 3e,f and it yields the exciton-free dead layer thickness in G1 as 1 nm and exciton free-layer dead layer thickness in G2 as 16 nm. The corresponding in-plane *n*_a_* = n_*a*_*+ ik*_*a*_ and out-of-plane *n*_c_* = n_*c*_*+ ik*_*c*_ optical constants are displayed in Figure S3. Note that the thick MoS_2_ characterized by the exciton-free dead layer exhibits transparent behavior along the *c*-axis, even at ultraviolet and visible wavelengths (usually in region of strong interband transition for semiconductors). Details of Fresnel modeling are provided in the Supporting Information.

**Figure 4:**
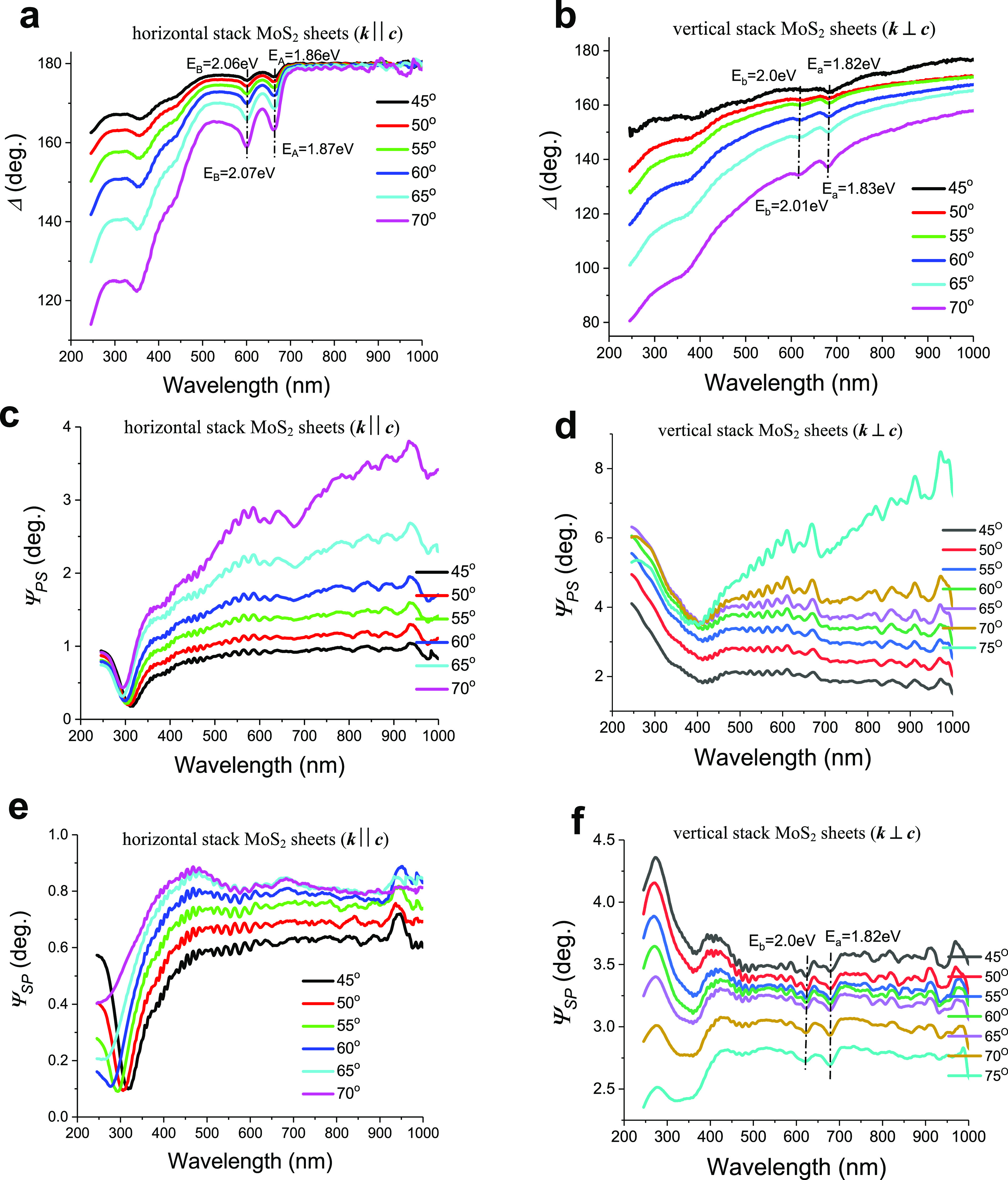
Anisotropy behaviors of excitons of horizontally/vertically stacked MoS_2_ sheets as a result of the polarization reflectance measurements for different angle of incidence. (a and b) Changes of phase Δ reflected light for two perpendicular sample orientations at various angles of incidence. (c–f) Off-diagonal ellipsometric angles *Ψ*_ps_ and *Ψ*_sp_ of the Jones matrix for reflected light, which corresponds to polarization mode conversion: from *p*(*s*) (c and d) to *s*(*p*) (e and f) polarization modes.

The change of reflection phase is more sensitive to the properties of materials and surfaces than that of reflection intensity,^33,34^ so detecting phase is more suitable for measuring the anisotropy of excitons in MoS_2_. Our experimental results show that the changes of ellipsometric phase Δ in the vicinity of exciton resonances are more pronounced than those of Ψ (Figure 4a,b). At a large angle of incidence (60°–70°), the ellipsometric data show the most significant contrast for measurement of phase changes Δ for two geometries G1 and G2 since the difference for the Fresnel reflection coefficients at flat sheet interfaces and their edges tend to maximal values (Figure 4a,b). In Figure 4c–f, the polarization conversions Ψ_ps_ (incoming *p*- into reflected *s*-polarized light) and Ψ_sp_ (vice versa) are shown. The off-diagonal terms of Jones matrix here refer to light that exits with a polarization orthogonal to the input polarization. Striking features in Ψ_ps_ and Ψ_sp_ are the disappearance in the reflectivity curves of the exciton doublet (A and B) for G1 at least for incident angles of 45°–60° (Figure 4c,e). Three major oscillators are involved in the measured spectral off-diagonal reflection Ψ_sp_ for G2 including the dips near A and B excitons and the high-energy C and D excitons (Figure 4f). At all incident angles, the Ψ_ps_ and Ψ_sp_ off-diagonal reflectivity components for G2 are larger than those for G1 (Figure 4c–f). Also, for G2, the Ψ_sp_ coefficient shows dips near A and B excitons while Ψ_ps_ does not. The reason for that is that the light before reflection can be projected onto the components in plane *ab* and perpendicular along the *c*-axis, which have different refractive indices. After reflection, this results in differing optical response between the components, which produces a composite reflected vector with both *p*- and *s*-components.^34^

### Mueller Matrix Measurements

**Figure 5:**
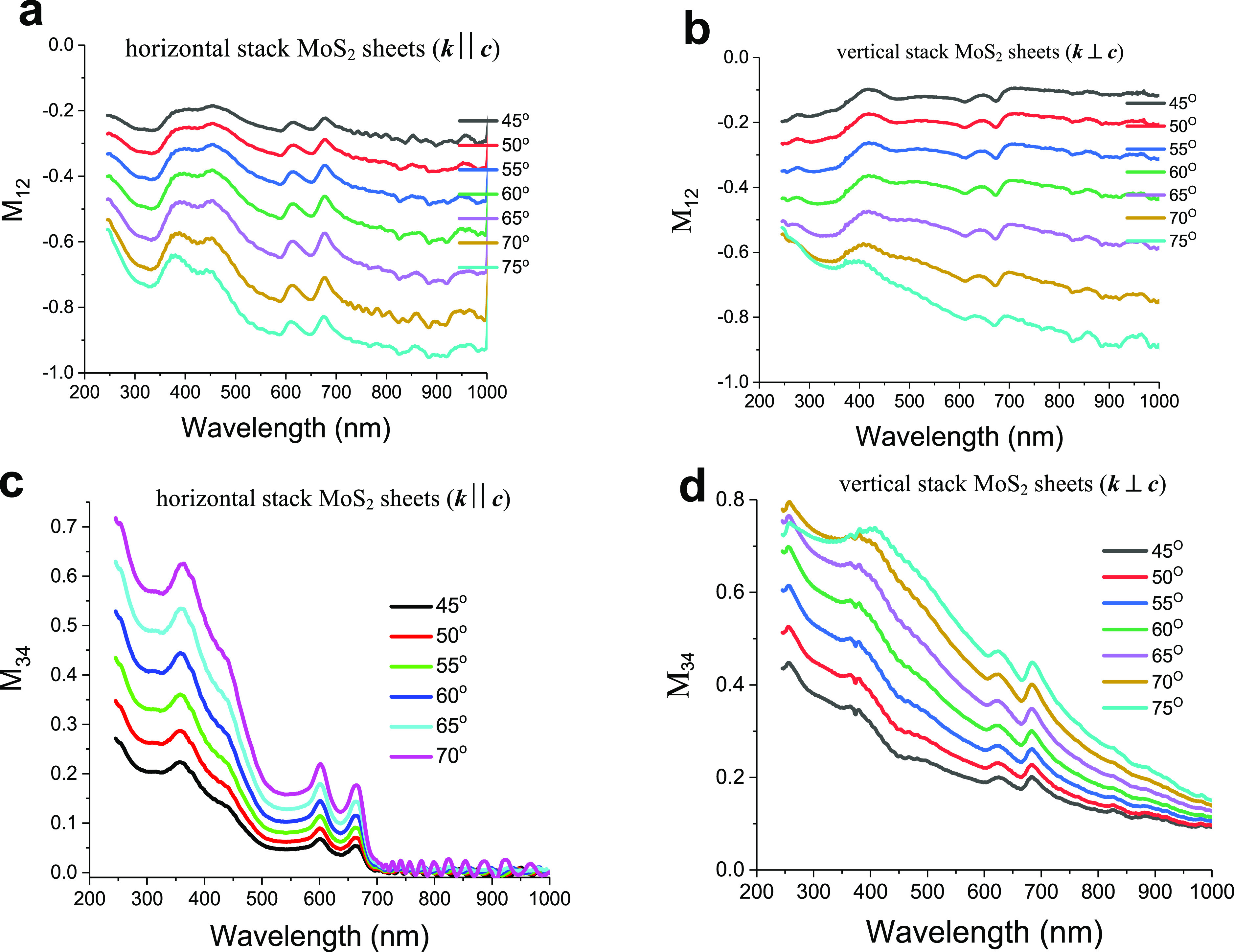
Optical anisotropy of horizontal and vertical stack of MoS_2_ sheets experimentally revealed by measuring the Mueller Matrix: (a and b) **m**_**12**_ and (c and d) **m**_**34**_.

In the next step, we experimentally investigated and compared Mueller matrix (MM) elements of the fabricated samples measured in G1 and G2 geometries. We found that the MM is block-diagonal, with the following relations: **m**_**12**_ = **m**_**21**_, **m**_**33**_ = **m**_**44**_, **m**_**34**_ = −**m**_**43**_, and, **m**_**22**_ = 1, suggesting that the sample is homogeneous. Quite interestingly, we observe that **m**_**13**_ spectra are quite similar to the cross-polarized reflectance spectra of Ψ_ps_ or Ψ_sp_ over the whole measurement range (**m**_**13**_ reflects the anisotropy effect of the sample, which strongly influences the polarization conversion). Note that off-diagonal elements of the MM are normally populated due to the presence of cross-polarization terms. We see that nondiagonal elements are nonzero for two different orientations G1 and G2, which confirms the optical anisotropy of stacked MoS_2_ sheets. Two of the 16 MM elements, **m**_**12**_ and **m**_**34**_, have been shown in Figure 5 as they possess the higher sensitivity to the different orientations of the sample. We can see that these Mueller matrix elements for the G1 sample orientation show the peaks at the excitonic transitions, while for G2 geometry, both matrix elements (**m**_**12**_ and **m**_**34**_) show dips at the excitonic transitions, compared with the spectra shown in Figures 2b and 3b. Figure 5 suggests, therefore, that it is also possible to distinguish G1 and G2 geometries through the spectral analysis of the matrix elements **m**_**12**_ and **m**_**34**_. It is worth reminding that the matrix element **m**_**34**_ usually provides an information about optical activity and birefringence of the sample (the birefringence of our samples can be described by *Δn = n*_*a*_ – *n*_*c*_, associated with the difference between the principal *n*_*a*_ (in-plane) and *n*_*c*_ (out-of-plane) refractive indices).^34,35^ Hence, the MM data also confirm the anisotropic properties of excitons in MoS_2_.

### Raman Spectra

In addition, the Raman spectra also demonstrate strong anisotropy of the phonon modes that depend on polarization of the light and direction of light propagation with respect to *c-*axis (see Figure S4, SI). We observe that the absence of optical absorption around the A and B excitons correlates with the pronounced strength of the Raman response for the in-plane mode (E_2g_) at ∼384 cm^–1^ and out-of-plane mode (A_1g_) at ∼408 cm^–1^ and additional Raman peaks at 287 and 493 cm^–1^ (Figure S4). Regardless of the sample orientation, the E_2g_ and A_1g_ modes intensities always peak for the both polarization aligned along the *a*-axis (*ab* sheet plane) and *c*-axis of the crystalline structure, because these modes involve primarily the out-of-plane atomic motions. Usually, the four principal frequencies of Raman spectra for horizontal stack of MoS_2_ sheets are ∼287, ∼384, ∼408, and 447 cm^–1^.^36,37^ The A_g_ mode with a frequency of 287 cm^–1^ reaches a maximum when the excitation polarization is parallel to the *c*-axis of the sample. Note that intensities of Raman signals are strongly dependent on local electrical fields on the surface of the sample. Moreover, the intensity ratio of the two main characteristic Raman peaks of MoS_2_ E^1^_2g_ and A_1g_, (*I*_E2g_/*I*_A1g_) decreases from ∼0.7 for G1 orientation (being approximately same for both polarizations) to ∼0.3 for G2 orientation. Such behavior of the ratio *I*_E__2g_/*I*_A1g_ correlates with the charge carrier density decreasing.^5,38^ Thus, the excitonic effects in stacked MoS_2_ sheets strongly regulate Raman scattering amplitudes and thereby explain the pronounced strength of the Raman response from the A and B excitons.

### FTIR Spectra

The infrared spectra of the horizontal stack of MoS_2_ sheets (G1 orientation) exhibit strong bands around of 633 and 763 cm^–1^ for both polarizations, arising from Mo—S vibrations (Figure S5). These modes are nondegenerate active modes of first order occurring due to in-plane vibrations normal to *c*-axis.^36^ For G2, high-frequency Mo—S vibration modes (600–750 cm^–1^) are vanished (see Figure S5b). The observed peaks at ∼1900–2000 and 3400 cm^–1^ are most probably connected to the C=C bonds and the stretching modes of OH groups present at the surface of MoS_2_ sheets.^39^

### Hopfield’s Model

To explain the properties of excitonic absorption for G1 and G2 sample orientations in MoS_2_, we have utilized the Hopfield and Thomas model.^14^ This model invokes spatial dispersion to describe optical properties of excitons. However, the presence of spatial dispersion requires an additional boundary condition to determine light reflection from a sample. Using Pekar’s ideas, Hopfield and Thomas elucidated the main features of this boundary condition (which turned out to be quite complex) and then simplified it by replacing the exciton potential energy near the surface by an infinite barrier with a finite distance *l* inside the crystal. As a result, they introduced a dead layer near the surface of the crystal, which is free from excitons (we stress an analogy of this approach with an introduction of a transitional layer that is used to describe surface effects for optical materials). The thickness *l* of the dead layer was assumed to be of the order of the size of the excitons^14^ (roughly speaking, this follows from impossibility for the center of mass of an exciton to be located less than an exciton radius closer to the surface). Hence, the light reflection from a semiconductor near excitonic transition in this simplified case is produced by the reflection from a thin exciton-free layer and the bulk of the semiconductor leading to interference effects that depend on the optical constants and geometry. It turned out that the simplified Hopfield’s model worked very well for many semiconductors, *e*.*g*., light interference effects explained evolution of excitonic peaks to excitonic troughs in CdSe.^15^ The extracted thickness of the dead layer *l* was found to be in the range from several nanometers to 100 nm depending on the exciton excitation level.^15^

In case of MoS_2_ (and other LMs), the anisotropy of excitons plays an important role. Due to anisotropic nature of dielectric constants of TMDCs, an exciton shape in MoS_2_ is elliptical with different sizes for the in-plane and out-of-plane directions. This implies that the exciton contribution to in-plane and out-of-plane response could be significantly different. Moreover, this also implies that thickness of the dead layer (free from excitons) is completely different for in-plane and out-of-plane excitons and, hence, for G1 and G2 geometries. The experimental data presented in Figure 2 suggest that the contribution of the dead layer to the exciton response is small in G1 geometry (the peaks stay the peaks), while contribution of the dead layer to the exciton optical response is large in G2 (the peaks become the troughs) (see Figure 3). Using Fresnel theory combined with the Hopfield model along with the anisotropic optical constants of MoS_2_ elucidated in ref (3) allows us to evaluate the thickness of the dead layer for both cases and hence the anisotropy ratio of MoS_2_ excitons. We have found that the maximal thickness of the dead layer that does not significantly change the reflection for G1 is around 1 nm, while the minimal thickness of the dead layer that will change the reflection maxima to reflection minima for G2 was 10 nm. This suggests that the exciton “shape” ellipse has the axes ratio larger than 10.

The theory evaluates the Bohr radius of the “interlayer” exciton (Figure 1a,d) in MoS_2_ as 1–3 nm,^40,41^ which is larger than the interlayer separation (0.6–0.7 nm) and is comparable with the values we got from fitting of optical spectra in G1 orientation. The theory also suggests that “intralayer” excitons (where electron and hole always reside on the same layer) are much wider and demonstrate a lower degree of correlation between the electron and hole.^42,43^ Overall, the weak van der Waals bonding along the *c*-axis in bulk MoS_2_ leads to strongly anisotropic excitonic properties. It was theoretically shown that the effective mass of carriers along the *c*-axis is much larger than that for motion in the *ab*-plane.^44^ Moreover, theoretical study revealed that the exciton wave function is confined mostly in individual S–Mo–S layers even in the bulk MoS_2_.^29^ Our observation of strong excitons and high-frequency Mo–S vibration modes coupling (600–750 cm^–1^) in horizontally stacked MoS_2_ sheets (see Figure S5) leads us to modify the Hopfield model of reflectance. We can assume that the A and B intralayer or interlayer excitons are either strongly or weakly bound to vibrational modes of Mo–S pairs of atoms that occupy the positions in the MoS_2_ hexagonal structure. The absence of vibrations at 633 and 763 cm^–1^ in the mid-IR range spectra for ***k*** ⊥ ***c*** orientation of sample strongly correlates with the vanishing of absorption by A and B excitons in the red spectra range and gives evidence of disappearance interlayer excitons due to the breaking of exciton–phonon coupling. Moreover, the Raman 287 cm^–1^ and IR 633, 763 cm^–1^ active phonon modes are enhanced by the A and B intralayer excitons, but the enhancement significantly decreases for interlayer-like excitons. However, observing the high-energy C exciton (∼2.6–2.7 eV) for the both orthogonal geometries (Figures 2 and 3) gives evidence that C excitons are mostly coupled to main E^1^_2g_ and A_1g_ phonon modes due to the symmetry-dependent exciton phonon interaction in MoS_2_. We found that the interlayer excitons are heavily damped in the case of their excitation with a wavevector of incident light directed perpendicularly to *c*-axis while the intralayer excitons are undamped. We can suggest that an overlap of the A and B excitons wave functions with adjacent atomic layers is negligible due to their large interlayer distance and weak van der Waals interaction. Thus, the anisotropic properties of excitons in layered MoS_2_ originate from competition between strong ionic/covalent bonds linking Mo and S atoms and out-of-plane links provided by weak van der Waals forces.

Finally, we note that actual surface conditions in the studied MoS_2_ crystals could be more complicated. Our experiments on nonpolished-virgin, roughly polished, high-quality polished, and LiOH treated samples (described in the Supporting Information, see Figure S2 and discussion) indicate that surface fields and damage may play some role in changing optical properties of the samples; however, they do not change the main results of our work.

## Conclusions

We have measured the anisotropic properties of light reflection from MoS_2_ samples for two orthogonal orientations with *c*-axis being perpendicular to the sample surface (the standard arrangement) and with *c*-axis being parallel to sample surface (reflection from the polished edge). We clearly see the anisotropy of exciton excitation in the second case, where the exciton peaks are observed only for incident light with the electric field being perpendicular to *c*-axis. While such anisotropy was well-established for photoluminescence, our measurements directly confirm the in-plane nature of A and B excitons in MoS_2_ from reflectance measurements.

We also found that the polarized reflectance line-shape and spectral position of A and B excitons for broad angles of excited light are critically dependent on the measured geometry chosen; moreover, the excitons do not contribute to optical response in the transitional layer (the dead exciton layer) in accordance to the Hopfield’s model. We show that the presence of the dead layer can significantly change the MoS_2_ reflection due to light interference. This implies that care is needed for extracting optical constants of MoS_2_ (and other LMs) from optical measurements especially for the case of thin flakes. Anisotropic ellipsometry measurements demonstrate the significant cross-polarization conversation (*s-* into *p-*polarization and vice versa) and decreasing of off-diagonal Mueller matrix elements **m**_**12**_ and **m**_**34**_ in the vicinity of A, B and C, D excitons for vertically stacked MoS_2_ sheets (*c*-axis parallel to the sample surface).

Our work could be useful for the development of tunable waveguides, optical modulators, and nonlinear devices based on TMDCs. As an important feature, oriented MoS_2_ crystals would allow one to improve the performance of exciton-based optoelectronic devices.

## Methods

### Fabrication of Samples

The standard horizontally stacked MoS_2_ sheets sample was fabricated by mechanically cleaving commercially available, natural bulk MoS_2_ (2D semiconductors) using the scotch tape method and transferring hundreds layer of MoS_2_ crystals onto a Si/SiO_2_ (290 nm)-substrate. Suitable layer crystals that were spatially isolated from bulk material were chosen for further device fabrication. The MoS_2_ crystals composed of two-dimensional layers MoS_2_ are stacked into the horizontal or vertical direction *via* van der Waals forces. Special geometry of sample can be regarded as an orderly vertically oriented stack of countless layers of MoS_2_ sheets along the direction of the *c*-axis (*i*.*e*., the optical axis of the MoS_2_).

The edges of vertically layered MoS_2_ sheets were polished using Ga ions technology and a focused ion beam (FIB) microscope (a hybrid FIB-SEM system, Carl Zeiss Crossbeam 540). For most cases, the 30 kV Ga ion beam was employed. On two occasions, the final cross-section polishing has been completed using 5 kV beams. Final polishing was made with the beam currents at 1.5 nA. The SEM images were obtained using two different detectors: in-lens and secondary electron detector (Figure 1d and Figure S1). The main problem was a very big size was required to mill out. Hence, we aimed to minimize the mill volume. Three samples have been fabricated. In the first sample W1, a rectangular slot of 150 μm length and 50 μm width to the depth of 60 μm was etched away using 100 nA mill. Then, the central area of 100 × 60 μm^2^ was polished with decreasing currents up to 1.5 nA. In the sample W2, a trapezoidal slot of 540 μm length and 60 μm width to the depth of 70 μm was etched away using 100 nA mill. Then, the central area of 230 × 70 μm^2^ was polished with decreasing currents up to 1.5 nA. In the last sample W3, a trapezoidal slot of ∼1500 μm length and (100–150) μm width to the depth of 250 μm was etched away using 80–100 nA mill. So as the system permits to etch only up to 600 μm in one direction, this has been split in three overlapping parts. Then, the central area of 280 × 250 μm^2^ was polished with decreasing currents up to 15 nA at 30 kV. The SEM image of the *ac*-plane from the freshly cleaved crystal after ions polishing reveals a local smooth surface, which ensures that our measurement results are reliable in view of the collection area of 100 × 200 μm^2^ (Figure 1c,f and Figure S1).

### Characterizations: Ellipsometric Measurements of MoS_2_ Layered Structures

The ellipsometry measurement was performed with a J.A. Woollam Co. rotating focused beam ellipsometer using variable angle spectroscopic measurements (VASE), in the range 240–1700 nm. The spot size on the sample was approximately 50 μm × 70 μm at ∼70°–80° angles of incidence. The ellipsometry measurement essentially monitors changes in the polarization state of incident light and the light reflected from the films. It yields two spectral parameters (Ψ and Δ) related with the amplitude (tan Ψ) and phase Δ of a complex reflectance ratio ρ, which indicates the ratio of the reflection coefficients for *p*-polarized (parallel to the plane of incidence) and *s*-polarized (perpendicular to the plane of incidence) light, ρ = *r*_p_/*r*_s_ = (tan Ψ) exp(*iΔ*), where *r*_p_ and *r*_s_ are the amplitude reflection coefficients for *p*- and *s*-polarized light.^33^ In addition to ellipsometric parameters Ψ and Δ, the ellipsometer allowed us to separately measure *R*_p_ = |*r*_p_|^2^ and *R*_s_ = |*r*_s_|^2^, the intensity reflections for *p-* and *s*-polarized light, respectively, at various angles of incidence with respect to the total light intensity.

Accurate determination of the complex refractive index and anisotropic properties of the stacked MoS_2_ sheets from spectroscopic ellipsometric data was also an objective of this study. To retrieve the optical constant from the measured results, we performed regression fitting using the Fresnel’s equations of a simple two-layer model that consists of thin MoS_2_ dead layer on top of a semi-infinite MoS_2_-like substrate (using WVASE32 software of J. A. Woollam Co., Inc.). We have also confirmed that the surfaces of all the films studied are atomically smooth (roughness <1 nm) by the SEM measurements (Figure 1 and Figure S1).

### Jones Matrix Measurements

A Woollam M2000F generalized ellipsometer allows to determine the diagonal and off-diagonal elements of the Jones matrix of fabricated MoS_2_ samples. In addition to measuring Ψ and Δ, our ellipsometer is also used to determine the generalized ellipsometric angles *Ψ*_ps_*, Ψ*_sp_ and *Δ*_ps_*, Δ*_sp_, which all links to the Jones reflection matrix.^33,34^ Off-diagonal ellipsometric functions *Ψ*_*ps*_ and *Ψ*_*sp*_ denote the part of reflected light which corresponds to polarization mode conversion: from *p*(*s*) to *s*(*p*) polarization modes. Incorporating cross-polarization effects into standard ellipsometry is the foundation of generalized ellipsometry.

### Mueller Matrix Measurements

To experimentally reveal MoS_2_ optical anisotropy for different stacked sample orientations, we measured the Mueller matrix (MM) using a Woollam M2000F spectroscopic ellipsometer in the polarizer-rotating compensator-sample-rotating analyzer-rotating arrangement. In this way, we have access to 16 normalized MM elements (traditionally MM normalized to **m**_**11**_, which is set to unity).^33,34^ The MM of a sample gives complete information about polarization properties of a sample under study; therefore, MM is a powerful and sensitive tool to fully characterize anisotropy and depolarization of samples, which cannot be achieved by simple intensity measurements. The MM is block-diagonal, and the symmetries (or antisymmetries) between the matrix elements are well-represented by the following relations: **m**_**12**_ = **m**_**21**_, **m**_**33**_ = **m**_**44**_, **m**_**14**_ = **m**_**41**_, **m**_**24**_ = **m**_**42**_ (identical elements pairs) **m**_**13**_ = −**m**_**31**_, **m**_**23**_ = −**m**_**32**_, **m**_**34**_ = −**m**_**43**_ (opposite element pairs) and, **m**_**22**_ = 1. This result suggests that the sample can be homogeneous, *i*.*e*., does not induce any type of depolarization. All the measured MM elements reflect the symmetry of the stacked MoS_2_ sheets samples with optical axes along α = 0° and 90° respect to *c*-axis of crystal.

### Raman and FTIR Characterizations

Polarized Raman and IR spectroscopy can be used to determine the orientation of the exfoliated anisotropic TMD crystals. The experimental setup used for Raman measurements was a confocal scanning Raman microscope, Renishaw. All measurements were carried out using linearly polarized excitation (*i*.*e*., perpendicular and parallel polarized) with wavelength 514 nm, 1800 lines/mm diffraction grating, and ×100 objective (N.A. = 0.90), whereas we used unpolarized detection to have a significant signal-to-noise ratio. The laser spot size was approximately 1 μm, and laser illumination power was about 0.5–0.7 mW, which cannot damage or oxidase the MoS_2_ layers.

Fourier transform infrared (FTIR) spectroscopy was performed in a Bruker Vertex 80 system with a Hyperion 3000 microscope. A variety of sources and detectors, combined with aluminum coated reflective optics, enable this system to be used from visible to mid-IR wavelengths. The measurement of the mid-IR reflection spectra was done at the normal incident at room temperature in the frequency range from 550 to 4000 cm^–1^ by performing 256 scans with a resolution of 4 cm^–1^ using cryogenic MCT detector cooled with liquid nitrogen. The measurements of polarized mid-IR spectra was performed with IR polarizer (A 675-P), as the reference were used the reflection from the gold thick mirror.

## Supporting Information Available

Discussions of fabrication details, measurement methods, characterization methods, and Fresnel modelling of samples and figures of SEM images, polarized reflectance spectra, optical anisotropy of MoS_2_, polarized Raman spectroscopy, and FTIR polarized spectroscopy (PDF)
