# Supplementary material for: “Dead” Exciton Layer and Exciton Anisotropy of Bulk MoS2 Extracted from Optical Measurements
Source: ACS Nano. 2022 Nov 9;16(11):18637–47. doi: 10.1021/acsnano.2c07169 (PMC9706669; doi:10.1021/acsnano.2c07169)
Supplement: Supplementary file 1 — nn2c07169_si_001.pdf [file nn2c07169_si_001.pdf]

Supplementary Information for

The "dead" exciton layer and exciton anisotropy of bulk MoS_2 extracted from optical measurements

Vasyl G. Kravets[†], Alexander A. Zhukov[‡], Matthew Holwill[‡], Kostya S. Novoselov[§], Alexander N. Grigorenko[†*]

[†] Department of Physics and Astronomy, Manchester University, Manchester M13 9PL, UK

[‡] National Graphene Institute (NGI), Manchester University, Manchester M13 9PL, UK

[§] Department of Materials Science and Engineering, National University of Singapore, Singapore 03-09 EA, Singapore

*corresponding author e-mail: sasha@manchester.ac.uk

Fabrication of samples.

The standard horizontally stacked MoS_2 sheet sample was fabricated by mechanically cleaving commercially available, natural bulk MoS_2 (2D Semiconductor supplies) using the scotch tape method [1] and transferring flakes of MoS_2 crystals onto a Si/SiO_2(290nm)-substrate. Flakes of suitable sizes that were spatially isolated from the bulk material were chosen for further device fabrication. The optical properties of the samples were measured when light was reflected from the top surface (with *c*-axis being perpendicular to the surface from which reflection was measured) and from the polished edge (with *c*-axis being parallel to the surface from which reflection was measured). The edges of as transferred vertically aligned MoS_2 flakes were rough (Figure S1a) which could affect the results of optical measurements due to scattering. To avoid the impact of roughness, edge segments (which were sufficiently large for optical measurements) were polished using Ga focused ion beam (FIB) microscope (a hybrid FIB-SEM system, Carl Zeiss Crossbeam 540). This was done using 30 kV Ga ion beam with current decreasing from 80-100mA in 3-4 steps down to 1.5nA. On two occasions (samples W1&W2, see below) the final cross-section polishing has been subsequently completed using 5kV beam at 1.5nA.

The SEM images of the measured surfaces were obtained using two different detectors: in-lens and secondary electron detector are shown in Figures 1d and S1. Three samples have been fabricated. In

the first sample, W1, a rectangular slot of 150 µm length and 50 µm width to the depth of 60 µm has been etched away using 100nA mill. Then the central area of 100x60 μm^2 was polished with decreasing currents up to 1.5nA. In the second sample, W2, a trapezoidal slot of 540 µm length and 60 µm width to the depth of 70 µm has been etched away using 100nA mill. Then the central area of 230x70 μm^2 was polished with decreasing currents up to 1.5nA. In the last sample, W3, a trapezoidal slot of ~1500 µm length and (100-150) µm width to the depth of 250 µm has been etched away using 80-100nA mill. As the system permits to etch only up to 600 µm in one direction, this has been split in 3 overlapping parts. Then the central area of 280x250 μm^2 was polished with decreasing currents down to 15 nA at 30kV. All samples showed the same optical features described in the main text.

Figure S1 show in situ images obtained by SEM at θ=54° tilt used for milling. Note that for the tilted sample the y-direction doesn't correspond to the scale bar and should be recalculated by sin(θ) and cos(θ) for the cross-section and sample surface, respectively. In particular, the thickness of the sample is 1/sin54° ≈ 1.236 bigger than obtained using the scale bar. SEM images of the *ac*-plane from the freshly cleaved crystal after ion polishing reveals a locally smooth surface, which ensures that our optical measurement results are reliable (with illuminated area being 100×200 μm^2), see Figs. 1e,f and Fig. S1.

Measurement methods.

As explained in the main text, the optical properties of samples were measured in two geometries: Geometry 1 (G1), where reflection from the top surface of the transferred flakes was measured (with *c*-axis being perpendicular to the surface from which reflection was measured) and Geometry 2 (G2), where the reflection from the polished edge of the flake was measured (with *c*-axis being parallel to the surface from which reflection was measured). For G1 at normal angle of incidence, the wave-vector of the light $\boldsymbol{k}$ is parallel to the unit vector $\boldsymbol{c}$ directed along the *c*-axis ($\boldsymbol{k} \parallel \boldsymbol{c}$ while $\boldsymbol{E} \perp \boldsymbol{c}$, where $\boldsymbol{E}$ is the electric field of the light wave). For G2 at normal angle of incidence $\boldsymbol{k} \perp \boldsymbol{c}$ while we have two different orientations of the electric field $\boldsymbol{E} \perp \boldsymbol{c}$ and $\boldsymbol{E} \parallel \boldsymbol{c}$. It is well known that layered MoS_2 crystal is built up of van der Waals bonded by S-Mo-S units. Each of these stable units consisting of two hexagonal planes of S atoms sandwiching a hexagonal plane of Mo coordinated through ionic-covalent inter-actions with each other in a trigonal prismatic arrangement [2,3] as schematically shown in Figure 1a,d. The anisotropy in the excitonic behaviours is attributed to crystal anisotropy and can be studied via the polarization dependent reflectance measurements. We found strong dependence of excitonic transitions A and B on polarisation of incident light for vertically stacked MoS_2 sheets (reflection from

the top surface of the fabricated flakes) as compared to that of the horizontally oriented sample (reflection from the polished edge of the fabricated samples). Optical anisotropy of MoS_2 flakes was studied by applying spectroscopic ellipsometry (SE) and Mueller–Jones matrix ellipsometry (MMSE) that yield complete characterization of an anisotropic sample. We employed a Woollam ellipsometer M2000F (J.A. Woollam Co., Inc.) with focusing system (NA = 0.1) that provides spatial resolution of 30*60 μm^2 on the sample surface. This instrument can measure the 16 normalized Mueller matrix elements and 4 Jones components [4]. In the Jones formalism for anisotropic materials, the off-diagonal reflection coefficient r_{ps} and r_{sp} (which correspond to the reflected *p*-polarised light induced by *s*-polarised light, and the reflected *s*-polarised light induced by incident *p*-polarised light[4]) are not zero. Variable angle ellipsometry routinely measures amplitude (Ψ) and phase (Δ) parameters for light reflected from a sample object at various angles of incidence (AOI), which are related to the reflected field amplitudes E_p and E_s for the incident light (E_i) of *p*- and *s*- polarizations, respectively, by the following equation $E_p / E_s = \tan(\Psi)\exp(i\Delta)$ [4]. In addition, our ellipsometer can measure intensity reflections and transmissions (R_p, T_p) for *p*- and (R_s, T_s) for *s*-polarised light (see Methods).

The edge measurements could be affected by the sample damage due to its polishing. To confirm that this is not the case and the main optical features observed in G2 geometry are not connected to polishing, we also measured reflection spectra from an unpolished flat edge of a suitable MoS_2 flake. The result of these measurements is shown in Fig. S2 and it confirms different behaviour of light reflection for two different polarizations: $\boldsymbol{E} \perp \boldsymbol{c}$ and $\boldsymbol{E} \parallel \boldsymbol{c}$. We can see that reflection from the edge of non-polished as-fabricated samples exhibit the same spectral shape as that observed for well-polished samples shown in Fig. 3a – the absence of exciton features for $\boldsymbol{E} \parallel \boldsymbol{c}$ (0° polarization) and exciton dips instead of exciton peaks for $\boldsymbol{E} \perp \boldsymbol{c}$ (90° degree polarization). Hence, the edge roughness and edge polishing did not result in significant qualitative differences in the reflection for two different orientations (while the absolute values of reflection did slightly change for non-polished and polished samples).

In Fig. S2c we compare the reflectance spectra for completely and partially polished stack of MoS_2 crystals at normal incidence and two polarisations of incident light. All spectral features of the *p*-polarised reflectance become slightly wider and spectral position of the B excitons is slightly blue shifted for the sample with only partially polished surface. At the same time, we observe no excitonic features for perpendicular polarization. Finally, Fig. S2d provides the optical reflection for 0° and 90° polarization for MoS_2 flake in G2 geometry with very rough surface. This sample showed unusual trend in the far-infrared range (where we observe an increase of reflection for this sample), yet it

confirmed the main features discussed in our manuscript: the absence of exciton features for $\boldsymbol{E} \parallel \boldsymbol{c}$ (0° polarization) and exciton dips instead of exciton peaks for $\boldsymbol{E} \perp \boldsymbol{c}$ (90° degree polarization).

It is worth noting that there is gradual transition from the absence of the excitonic peaks to the excitonic dips under gradual change of the polarization (Figure S2e) with absence of polarization related features for photon energies below the exciton energy 800-1100 nm. We also checked that treatment of the polished sample with a dilute solution of LiOH (in order to remove possible effect of stress) did not change the reflection properties from the sample edge. However, LiOH treatment of the samples could lead to intercalation of some amount of Li atoms into MoS_2 flakes changing the absolute value of polarised reflectance. Again, we found that the main qualitative features of the measured optical spectra remain the same (the absence of excitonic peak for 0°-polarization and dips instead peaks for 90°-polarization), see Fig. S2f.

The absence of excitonic features for 0°-polarization as well as the change of the peaks to dips for 90° polarization cannot be simply described by the Tauc-Lorentz oscillator model [5,6]. This requires strong anisotropy of exciton properties as well as the introduction of the "dead" exciton layer suggested by Hopfield and Thomas [7]. Our experiments on non-polished-virgin, high quality polished and LiOH treated samples indicate that while surface damage and scattering may play some role in explaining optical feature near exciton transitions, the "dead" exciton layer is the most straightforward explanation of our results. Indeed, adding "dead" exciton layers of different thicknesses for the two geometries (G1 and G2) in Fresnel modelling and performing simultaneous fitting of the measured reflections in the two geometries (G1 and G2) – which can be done using Wvase software – we can extract the thickness of the "dead" layers. This fit is shown in Figure 3 e,f and it yields the exciton-free "dead" layer thickness in G1 as 1nm and exciton free-layer "dead" layer thickness in G2 as 16nm. The corresponding in-plane $n_a^*=n_a+ik_a$ and out-of-plane $n_c^*=n_c+ik_c$ optical constants extracted form the modelling are presented in Figure S3. Note that the thick MoS_2 characterized by the exciton-free "dead" layer exhibits transparent behaviour along the *c*-axis, even at ultraviolet and visible wavelengths (usually in region of strong interband transition for semiconductors).

Raman spectroscopy. Polarised Raman spectroscopy can be used to determine the orientation of the exfoliated anisotropic MoS_2 crystals. Raman measurements were performed using a confocal scanning Raman microscope, Renishaw. All measurements were carried out using linearly polarized excitation (i.e., perpendicular and parallel polarized) with wavelength 514 nm, 1800 lines/mm diffraction grating, and ×100 objective (NA = 0.90). The laser spot size was approximately ~1 μm in diameter. The laser power was less than 0.5-0.7 mW for which no physical damage or oxidation was expected to occur in

the studied MoS_2 flakes. Bulk molybdenum disulphide MoS_2 belongs to the class of transition metal dichalcogenides (TMDs) that crystallize in the characteristic 2H polytype. The corresponding Bravais lattice is hexagonal and the space group of the crystal is D_{6h}^4, where the repeat unit in the *c* direction contains two layers and the S atoms in one layer are directly above the Mo. The unit cell is characterized by the lattice parameters *a* (in-plane lattice constant) and *c* (out-of-plane lattice constant) [8]. According to the group theory, for a perfect MoS_2, the normal vibration modes at the centre of the Brillouin zone can be expressed as [9]

$$\Gamma(MoS_2)=A_{2u}(IR) + E_{1u}(IR) + A_{1g}(R) + 2E_{2g}(R) + E_{1g}(R).$$

In the above expression A_{1g}, E_{1g}, and E_{2g} are the Raman active modes while A_{2u} and E_{1u} modes are infrared active. The four principal frequencies of Raman spectra for horizontal stack of MoS_2 sheets are ~287, ~384, ~408 and 447 cm^{-1} for both polarisations of excited light (see Figure S4a). In the case of 90°-polarisation excited light the Raman spectra for vertical stack of MoS_2 sheets mainly reproduces the features of Raman spectra for previous orientation. In contrast, the Raman spectra for 0°-polarisation two peaks at ~287 and 447 cm^{-1} vanishes but appears the new maximum at 493 cm^{-1}. Note that the observed peaks are not consistent to conventional Raman peaks of MoS_2 and the intensity ratio between the E^1_{2g} and A_{1g} Raman most active modes (I_{E2g}/I_{A1g}) changes from ~0.7 for the ***k*** II ***c*** orientation (approximately the same for both polarisations) to ~0.3 for the ***k*** ⊥ ***c*** geometry of measurements. The intensity of A_{1g} mode with a frequency of 408 cm^{-1} reaches a maximum when the excitation laser is perpendicular to the *c*-axis of the sample. Additionally, the Raman spectroscopy also demonstrates that intensity of phonon modes and half width of peaks for two different orientations of sample change with various polarization of the excited light and again depends on orientation of the sample (Figure S4).

FTIR spectroscopy. Fourier Transform Infra-Red (FTIR) spectra were collected in air, with a Bruker Vertex 80 spectrophotometer, equipped with a Hyperion 3000 microscope and a nitrogen cooled MCT detector for middle IR region. Each spectrum was collected at the closed to normal incident (θ<12°) at room temperature by using 256 scans with a resolution of 4 cm^{-1}. The measurements of polarised mid-IR spectra was performed with IR polarizer (A 675-P), as the reference was used the reflection from the gold thick mirror. In the mid-IR region 550-4000 cm^{-1} feature arises due to vibrational modes of Mo–S pairs of atoms that occupy the positions in the MoS_2 hexagonal structure and the vibrations of Mo atoms with respect to S related vacancies. Due to limitation of the spectral range of our FTIR spectrometer, we cannot observe Reststrahlen band of MoS_2: the longitudinal (LO), ω_{LO}~470 cm^{-1} and transverse (TO), ω_{TO}~384 cm^{-1} optical phonon modes. However, the spectra of the horizontally stacked

MoS_2 sheets (Figure S5a) for both 0°- and 90°-polarisations are characterised by the vibrational manifestations of the narrow and strong bands at 633 and 763 cm^{-1}, which is the infrared active vibration of Mo-S [10,11]. These vibrations may be assigned to the combinations and overtones of fundamental modes of active IR modes in bulk MoS_2 [10]. Remarkably, vibrations at 633 and 763 cm^{-1} in the mid-IR range spectra are vanished for $\boldsymbol{k} \perp \boldsymbol{c}$ geometry of orientation. Thus, the fine structure of the vibrational spectrum in the 600–800 cm^{-1} range for the $\boldsymbol{k}$ II $\boldsymbol{c}$ sample/light orientation is another signature of the activity of vibration Mo-S witch correlates with appearance of A and B excitons in visible (red) spectral range (Figure 2a,b). In accordance to Ataca *et al.* [10], the weak absorption in the 1150–1000 cm^{-1} range observed in the IR spectra (Figure S5b) for $\boldsymbol{k} \perp \boldsymbol{c}$ geometry is mostly due to sulfate groups adsorbed on defect sites. This indicates that virgin MoS_2 contains some adsorbed impurities, presumably formed by oxidation of the surface and likely occurring at defect sites of edges. Thus it was shown that strong anisotropy persists up to phonon modes of the mid-IR region in MoS_2 [9]. The observed additional features at ~1900-2000 and 3400 cm^{-1} probably correspond to the C=C and O-H bonds present at the surface of MoS_2 sheets.

Fresnel modelling of samples.

Wvase32 software of J. A. Woollam Co., Inc., was used for modelling. The software employs Fresnel theory in order to calculate the reflection/transmission coefficients from layered samples with given optical constants. It also allows one to restore optical constants of an unknown layer placed on a given substrate from experimental measurements of ellipsometric parameters. For isotropic samples, measurements of ellipsometric parameters Ψ and Δ at one angle of incidence is normally enough to extract the values of *n* and *k* of an unknown layer. For anisotropic samples, variable angle ellipsometry is used to extract optical constants in which ellipsometric reflections of an unknown sample are simultaneously fitted for several different angles of incidence.

Figure 2d shows the fitting of the Ψ spectra (the Δ spectra were fitted simultaneously) of thick MoS_2 sheets in G1 geometry at several different angles of incidence with the help of Wvase32 software. In this modelling, MoS_2 was assumed to be bi-anisotropic with different optical constants for in-plane and out-of-plane direction (MoS_2 was also assumed to be semi-infinite due to large absorption). One can notice an excellent agreement of the fitted data with the experimental data. The in-plane constants are shown in Fig. 2e. Using these constants and the Wvase32 software, we calculated the reflectance

for the samples in G2 geometry and found that it cannot explain the measured experimental data, see Fig. 3d where we obtained peaks in the modelling data and dips in the measured data.

To address this disagreement, we have simultaneously modelled the measured ellipsometric spectra of Ψ and Δ in G1 and G2 geometries acquired under several angles of incidence by adding an additional "dead layer" with different thicknesses for G1 geometry and G2 geometry (which Wvase32 software is capable of). The thickness of the "dead layers" in G1 and G2 geometries was a fitting parameter (yielding ~1nm thickness for G1 geometry and ~10nm for G2 geometry) while the optical constants of the "dead layer" were chosen to be the bi-anisotropic optical constants of MoS_2 shown in Fig S3 with excitonic peaks being removed. Figure 3e and f shows the results of the modelling.

References

(1) Novoselov, K. S.; Jiang, D.; Schedin, F.; Booth, T. J.; Khotkevich, V. V.; Morozov, S. V.; Geim, A. K. Two-dimensional atomic crystals. *Proc. Natl. Acad. Sci. U.S.A.* **2005**, *102*, 10451.

(2) Geim, A. K.; Grigorieva, I. V.; Van der Waals heterostructures, *Nature* **2013**, *499,* 419-425.

(3) Novoselov, K.S.; Mishchenko, A.; Carvalho, A.; Castro Neto, A.H. 2D materials and van der Waals heterostructures. *Science* **2016**, *353*, 6298.

(4) Azzam, R. M. A.; Bashara, N.M. *Ellipsometry and Polarized Light*, 1st ed.; North-Holland: Amsterdam, 1977.

(5) Ramasubramaniam, A. Large excitonic effects in monolayers of molybdenum and tungsten dichalcogenides. *Phys. Rev. B* **2012**, *86*, 115409.

(6) Kravets, V. G.; Wu, F.; Auton, G. H.; Yu, T.; Imaizumi, Sh.; Grigorenko, A. N. Measurements of electrically tunable refractive index of MoS_2 monolayer and its usage in optical modulators. *npj 2D Materials and Applications* **2019**, *3*:36; https://doi.org/10.1038/s41699-019-0119-1.

(7) Hopfield J. J.; Thomas, D. G. Theoretical and Experimental Effects of Spatial Dispersion on the Optical Properties of Crystals. *Phys. Rev.* **1963**, *132*, 563.

(8) Molina-Sánchez, A.; Hummer, K.; Wirtz, L. Vibrational and optical properties of MoS_2: From monolayer to bulk. *Surface Science Reports* **2015**, *70*, 554–586.

(9) Wieting, T. J.; Verble, J. L. Infrared and Raman Studies of Long- Wavelength Optical Phonons in Hexagonal MoS_2. *Phys. Rev. B* **1971**, *3*, 4286−4292.

(10) Ataca, C.; Topsakal, M.; Aktürk, E.; Ciraci, S. A Comparative Study of Lattice Dynamics of Three- and Two-Dimensional MoS_2. *J. Phys. Chem. C* **2011**, *115*, 16354−16361.

(11) Cai, Y.; Lan, J.; Zhang, G.; Zhang, Y.-W. Lattice Vibrational Modes and Phonon Thermal Conductivity of Monolayer MoS_2. *Phys. Rev. B* **2014**, *89*, 035438.

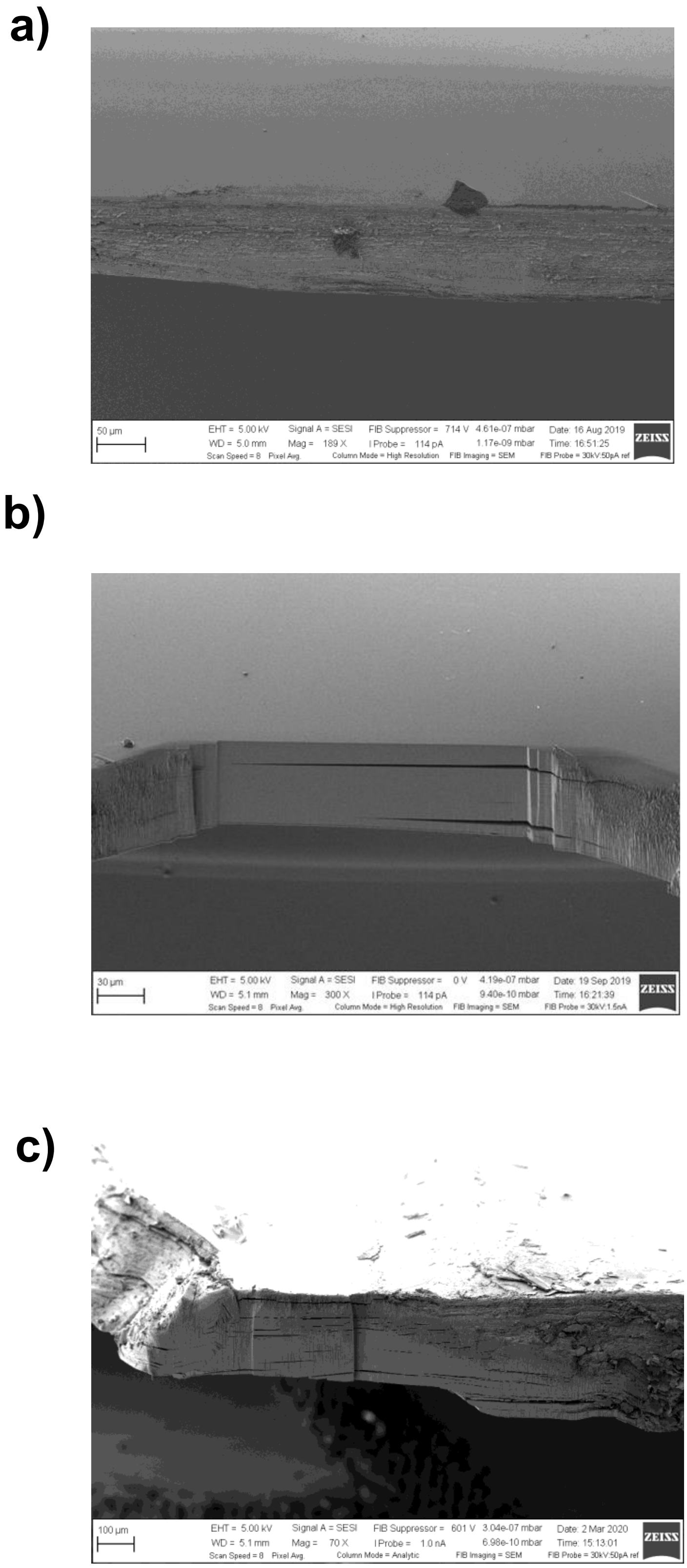

Figure S1. Scanning electron microscopy (SEM) images of vertical stack of MoS_2 sheets before and after focused ion beam (FIB) milling: **a)** Pristine surface of sample W2 edge before FIB milling. **b)** Polished surface of W2 edge after final FIB milling. **c)** Polished surface of W3 after final FIB milling.

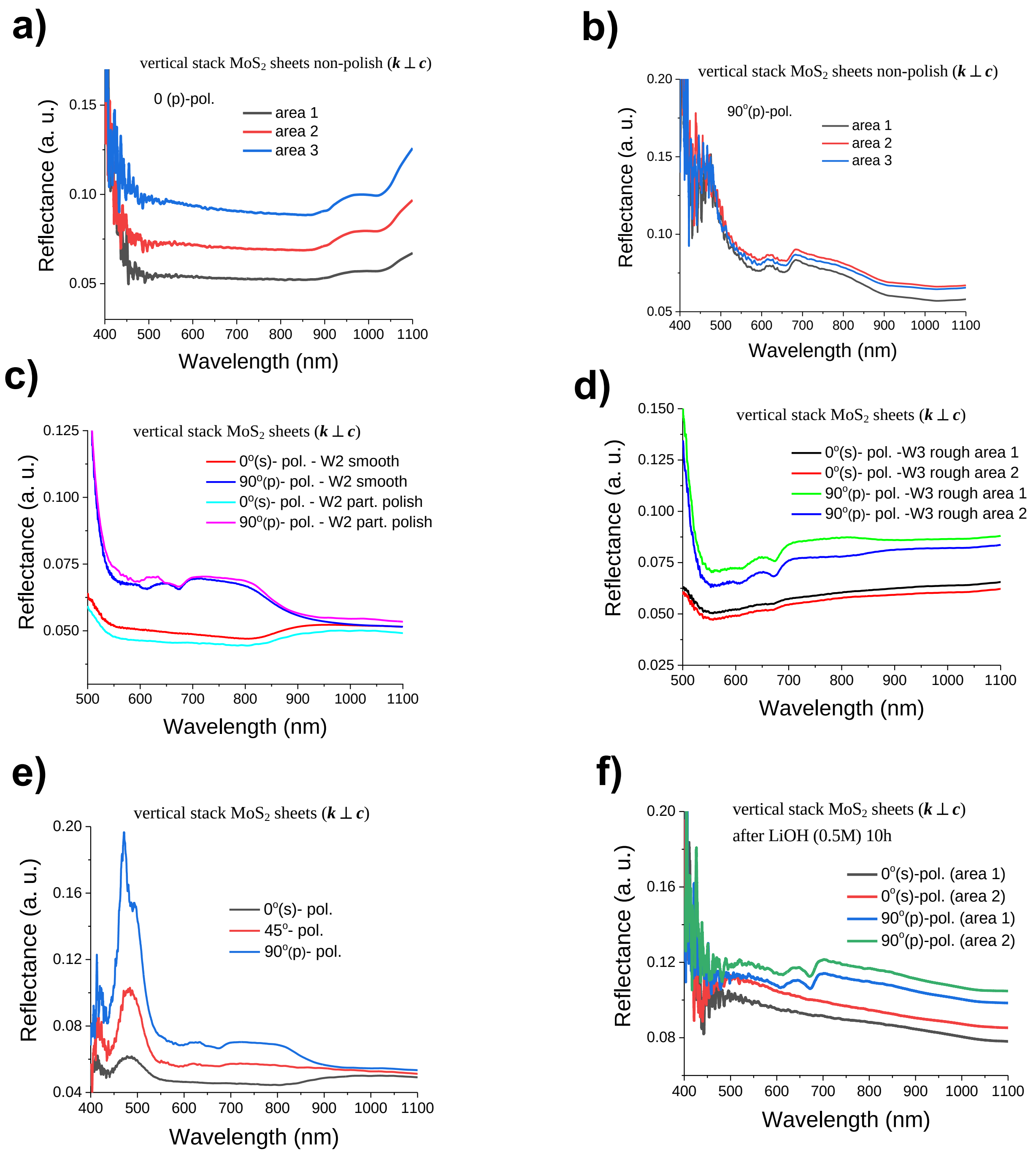

Figure S2. The polarised reflectance spectra for vertically stacked MoS_2 sheets (***E*** ⊥ ***c***, ***E*** II ***c***; ***k*** ⊥ ***c***). (a,b) Reflectance spectra for non-polished stack of MoS_2 sheets at normal incidence and two polarisations of incident light. (c) Reflectance spectra for completely polished and partially polished stack of a MoS_2 crystal at normal incidence and two polarisations of incident light. (d) Reflectance spectra for polished stack of MoS_2 sheets at normal incidence and two polarisations of incident light. (e) Reflectance spectra influenced by the absorbance of the A and B excitonic bands as well as interband transition for vertical stack of MoS_2 sheets as a function of polarisation angles. (f) Reflectance spectra for stack of MoS_2 sheets after treatment of sample in LiOH (0.5M, 10 hours) at normal incidence and two polarisations of incident light.

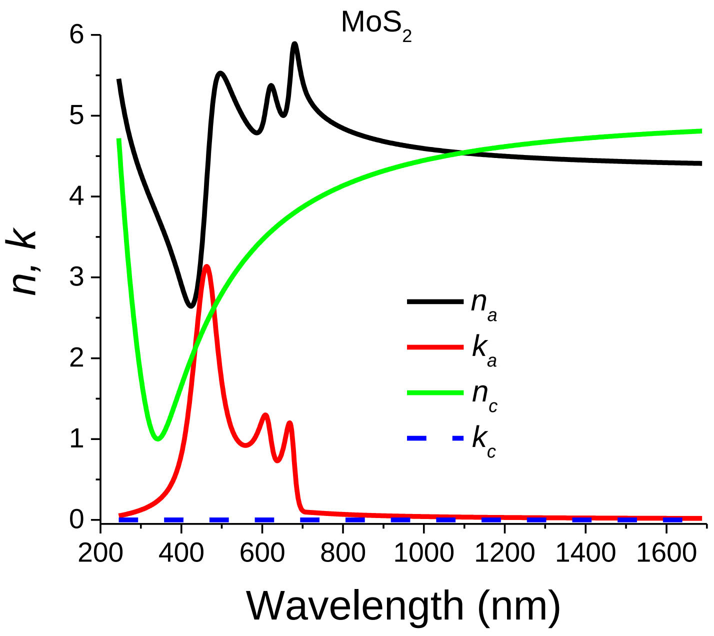

Figure S3. Optical anisotropy of MoS_2. Real *n* and imaginary *k* parts of the optical constants in-plane (n_a, k_a) and out-of-plane (n_c, k_c).

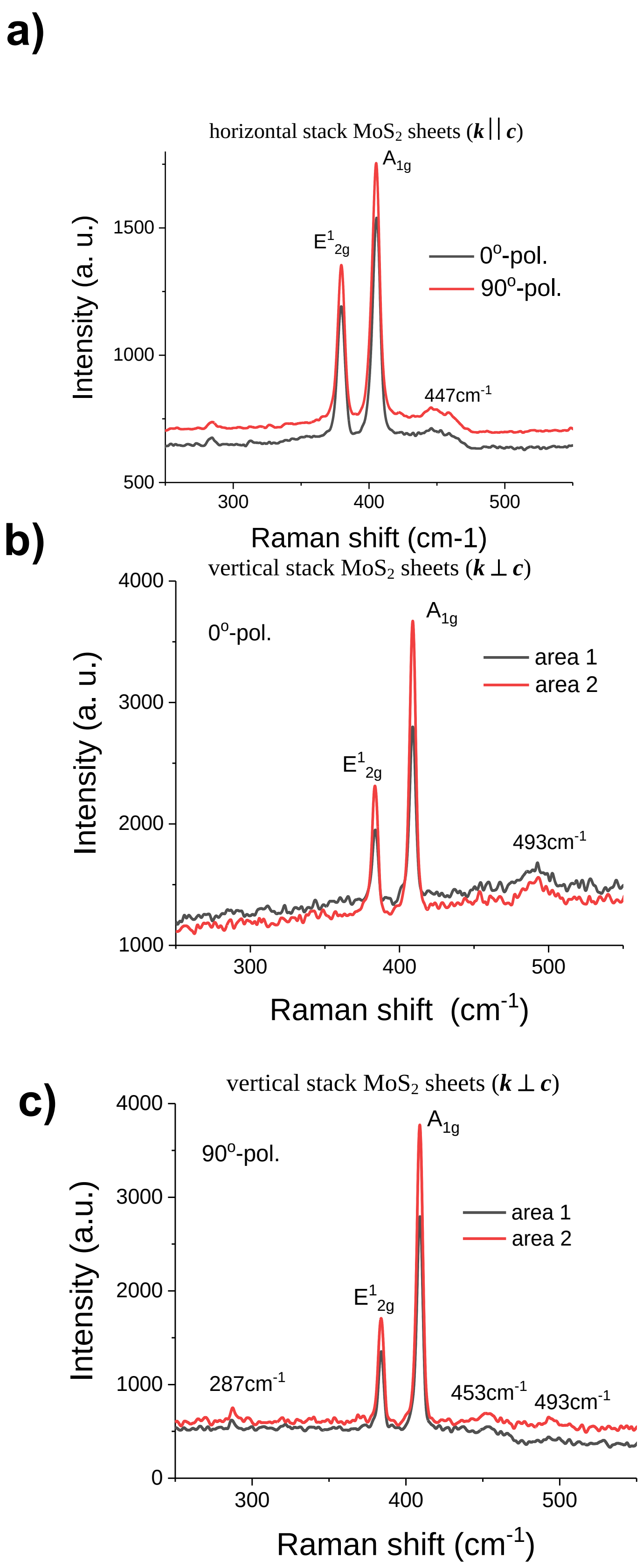

Figure S4. Polarised Raman spectroscopy of stacked MoS_2 sheets excited with λ_{exc}=514.5nm. **(a)** Raman spectra of main features for horizontal stack of MoS_2 sheets taken at parallel and perpendicular polarizer angles from the two sample areas. **(b,c)** Raman spectra of main features for vertical stack of MoS_2 sheets taken at parallel **(b)** and perpendicular **(c)** polarizer angles from the two sample areas.

a)

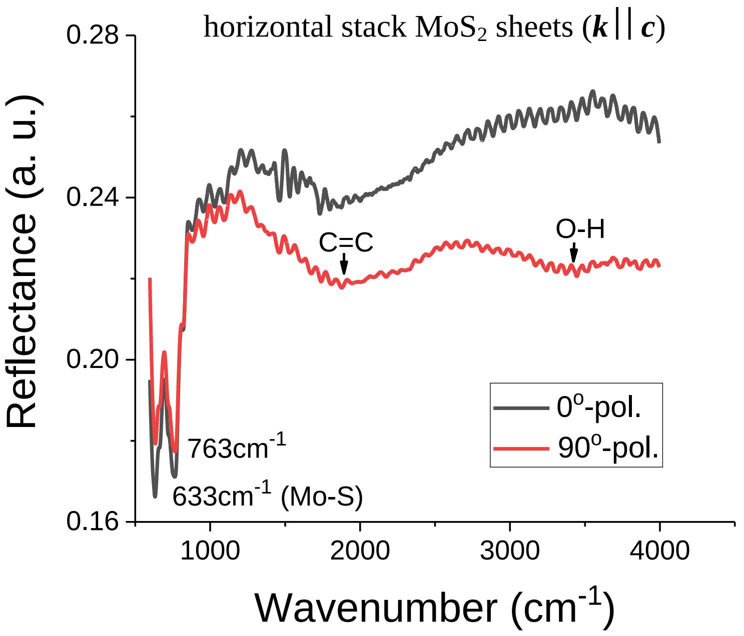

b)

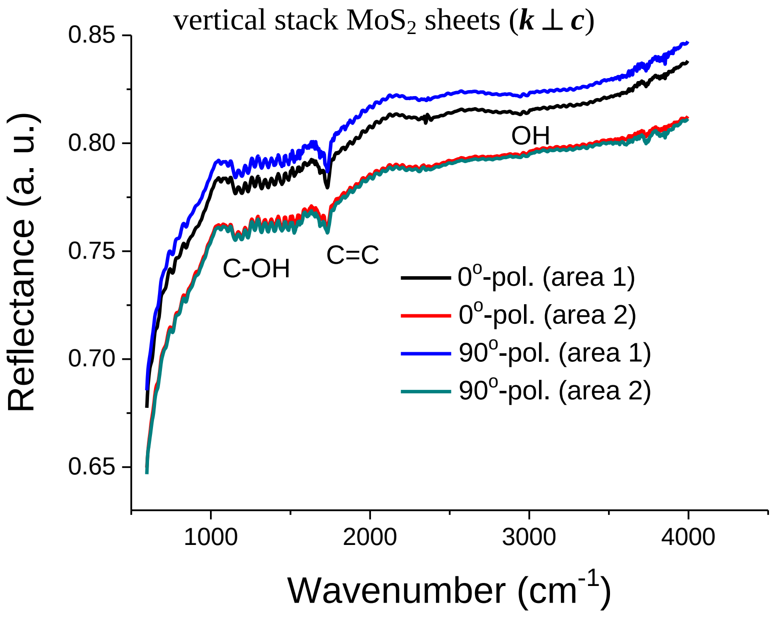

Figure S5. The FTIR polarised spectroscopy of stacked MoS_2 sheets. **(a)** Mid-IR spectra for horizontal stack of MoS_2 sheets taken at parallel and perpendicular polarizer angles from the sample. **(b)** Mid-IR spectra of main features for vertical stack of MoS_2 sheets taken at parallel and perpendicular polarizer angles from the two sample areas.
